# Return to the New Normal: Empirical Analysis of Changes in E-Consumer Behavior during the COVID-19 Pandemic

**DOI:** 10.3390/bs12030085

**Published:** 2022-03-18

**Authors:** František Pollák, Peter Markovič, Roman Vavrek, Michal Konečný

**Affiliations:** 1 Faculty of Corporate Strategy, Institute of Technology and Business in České Budějovice, Nemanická 436/7, 370 10 České Budějovice, Czech Republic; michal.konecny@mail.vstecb.cz; 2 Faculty of Business Management, University of Economics in Bratislava, Dolnozemská cesta 1/b, 85235 Bratislava, Slovakia; peter.markovic@euba.sk; 3 Department of Public Economics, Faculty of Economics, VSB—Technical University of Ostrava, Sokolská třída 33, 702 00 Ostrava, Czech Republic; roman.vavrek@vsb.cz

**Keywords:** consumer behavior, social networks, Facebook, COVID-19, social distancing

## Abstract

The global pandemic caused by the new coronavirus has largely changed established business practices. The aim of this study is to present the results of eighteen months of intensive research into the effects of the pandemic on e-consumer behavior. In one of the most active e-commerce markets in Europe, the Czech Republic, we analyzed a sample of more than one and a half million Facebook users in terms of their C2B interactions on the B2C activities of the five major e-commerce market players. The measurements were carried out in three periods, which corresponded to the onset of the first wave, the peak, and the fading of the second wave of the pandemic. This enabled us to monitor the effect of seasonality and the stabilization of patterns of consumer behavior during the coronavirus crisis. The results suggest that a specific panic pattern of e-consumer behavior was developed at the time of the onset of the pandemic. However, as the pandemic progressed, the market adapted to a new normal, which, as evidenced by the change in trends, appears to be a combination of the pre-pandemic and pandemic behavioral patterns. Using a statistical analysis, it was possible to identify the delta of changes within the patterns of consumer behavior, thus fulfilling the final condition for creating an empirical model of the impacts of the COVID-19 pandemic on e-consumer behavior presented in this study.

## 1. Introduction

The first decade of the twenty-first century was marked by the accelerated digitization of processes within traditional business. Businesses gradually switched to online sales, even in areas that had only recently been the domain of the classic world of brick-and-mortar stores [1,2,3]. The demand side of the market, represented mainly by final consumers, benefited from the opportunities provided by online technologies. The priority was to gain benefits in terms of lower prices of goods; the secondary motivation was to satisfy higher demand [4]. However, the digital ecosystem was only an additional sales channel.

The first months of 2020, during which most market-oriented countries introduced forced closures, set a significant precedent for the continuity of e-market development [5,6]. In the effort to slow down the pandemic, traditional brick-and-mortar shops were closed almost overnight, making the online environment an affordable alternative for the safe procurement of demanded goods and services [7]. Information and communication technologies, and especially m-commerce tools, became interactive gateways to businesses [6].

In the Czech Republic, during the first days after the introduction of the state of emergency, sales in a significant part of the business sector decreased considerably; companies using the O2 eKasa payment solution saw a drop of one-tenth of their normal turnover. In terms of growth, only the turnover of payments made by m-commerce instruments grew in the period under review; more precisely, there was a 20% increase compared to the average values [8]. Even the most important shops in the traditional world, such as petrol stations, saw significant revenue shortfalls, as they literally had to fight with the growing lack of drivers, despite the ever-decreasing oil prices [9]. As for traditional offline players in the market, nearly a 100% drop in the volume of sales affected, e.g., jewelry retailers, and catering and accommodation services saw a drop in sales of well above 90% [10]. Buying behavior patterns began to change under the pressure of circumstances [11]. In a panic response to the situation, consumers began to focus on the accumulation of daily consumer goods, which was roughly the same regardless of the geographic location of the demand market [12]. In terms of the nature of the products, they differed only slightly. The dominant factor in deciding on the type and nature of products was mainly the economic situation of the buyer [13]. The market situation changed not only the patterns of consumer behavior, but also the overall perception of consumer life from the holistic perspective. Consumers began to consider the impacts of their purchases either on their health or on the environment as such [14]. Social distancing combined with working from home accelerated these changes. From the point of view of the pace of change, we could see a significantly accelerated, even revolutionary trend. Changes that would otherwise take years were implemented into the lives of individuals within a week in the conditions of the forced global closure of shops due to the pandemic. At this point, we, as researchers, decided to build on our past projects [4,15] and focus our research capacities on analyzing the impact of the COVID-19 pandemic on e-consumer behavior. In the initial phase, we implemented pre-research qualitative data collection (visualized and described in more detail in the next chapter), which determined the basic methodological framework for the research. This phase was followed by the first measurement, or the first collection of empirical data.

As the pandemic progressed, its impact on consumer behavior took on new dimensions, and Euromoney Country Risk [16] conducted a global study to assess the risks of the possible next waves to national economies, with the Czech Republic’s predicted risk being beyond the averages of similar countries. At the same time, the risk profile of the Czech Republic deteriorated the most out of all European countries. Together with the relatively vague prognosis of the pandemic, this has only multiplied a number of factors that need to be taken into account in a comprehensive view of the situation. During this period, however, it was possible to see an indication of stabilizing trends. According to Nielsen’s retail audit [17], after the spring wave, which was also characterized by panic buying, the situation stabilized in the summer. The stabilization had the form of a one-tenth decrease in shopper turnout, while online sales increased by more than 20% in the previous year. In a year-on-year comparison within the quarantine months, this was a 101% increase. The new summer normal of the online store represented a stabilization of the trend at +50% compared to the previous year.

With the onset of the second wave, this increase increased to +91%. The trend of panic buying declined as consumers that learned from the first wave realized that supply disruptions were highly unlikely [18,19]. These facts were also reflected in the e-consumer behavior of the examined sample during the second measurement, in which both the regional specificity parameter and the seasonality parameter were tested.

With the aftermath of the second wave of the pandemic in the first half of 2021, it was possible to proceed with the research of the context, as the empirical material collected during the two measurements showed a high degree of specificity. In order to confirm the assumptions, the third and final collection of empirical material was carried out (described in more detail and visualized in the following chapter). Within the study of the current state and the continuous deepening of knowledge in the issue, several assumptions made in the previous phases of the research were confirmed. These were mainly the assumption of stabilized online consumer habits [20,21], preference for online shopping over traditional ways of shopping [22,23,24], preference for working from home [25], and the digitization of the offer of hitherto dominant offline products [26,27,28]. In general, we can see the stabilization in both the supply and demand. Both sides of the market have been able to adapt to the new situation [29,30]. Regarding experts’ view of trends across the global market, the TOP 10 Global Consumer Trends study [31] presented a comprehensive and integrated study that particularly pointed out the emergence of a trend towards sustainability, desire for comfort, or digital reality and time flexibility. The last of these trends is important from the perspective of the study being submitted.

Based on the results of our own research [32,33], in the first phase of our research, patterns of consumer behavior were identified that showed panic behavior; given the situation, it was a relatively logical and predictable fact. Consumer behavior in times of uncertainty is specific, as evidenced by several studies [34]. In general, it manifests itself mainly in the accumulation of goods and the creation of stocks. This activity of customers can be considered rational in some respects [35], as it is based on the need to prevent objective shortages. In any event, such consumer behavior has a dominant negative impact on supply chain stability [36]. As for panic behavior, it was not just the demand-side domain [37]; companies showed a high degree of improvisation in the first months of the pandemic as well. Regarding the geographical specificity of the patterns of behavior, in the reference studies, we recorded relatively similar panic patterns of behavior in terms of customer reactions to the same or similar external stimuli caused by the COVID-19 pandemic [38,39,40]. Through the synthesis of knowledge, it was possible to describe the panic pattern of pandemic e-consumer behavior as a condition, where under the pressure of circumstances, consumers maximized their consumption by seeking additional benefits through online consumer-to-business (C2B) activities. Both sides of the market found space for interaction in the virtual environment, where social networks—in our case, especially the social network Facebook—replaced the traditional meeting spaces in the times of social distancing [41,42,43]. As is well known from reference research [44,45], in the environment of Facebook, business entities carry out their e-marketing activities with a business-to-consumer (B2C) nature. They regularly communicate their e-marketing messages to target markets represented by fans of their profiles [46]. It was these interactions and their metadata that provided the empirical material for statistical processing.

Despite the high specificity of seasonality or regional specificity, the trends stabilized at the turn of 2020/2021 [47].

At this point, we come to the definition of a basic scientific problem that needs to be clarified before an empirical model of the effects of a pandemic on e-consumer behavior can be finalized. Specifically, it is a definition of the degree of change in behavior across the various stages of the pandemic. The research problem is converted into a research question as follows:

*How has the COVID-19 pandemic affected e-consumer behavior in the monitored market?*

To solve the research problem, the market is represented by both the supply and demand side. The demand side consists of consumers/users of the social network Facebook. The supply side consists of the main representatives of the Czech e-commerce market, represented by their profiles in the form of fan pages.

From the point of view of the structure of the study, the following chapters will build on the introduction, which synthesizes and presents the reference resources in chronological order. The theoretical framework was complemented by the results of our research [4,15,32,33,47]. The aim of the synthesis was to define a research problem and formulate research questions. Based on this framework, we will further describe the methodology, which will be complemented by the visualization of individual research phases. The research results are presented and discussed in the next chapter. Finally, an empirical model is completed, visualized, and described as a final output of eighteen months of efforts of the authors to find an answer to the research question. In the conclusion, the results of the research are evaluated and the basic implications for science and practice are specified.

The present study is the final output of a comprehensive study of the impacts of the COVID-19 pandemic on e-consumer behavior.

## 2. Materials and Methods

The main goal of this study is to identify changes in the interactions of digital customer communities of selected representatives of electronic businesses on the Czech market during 3 observation periods carried out during the COVID-19 pandemic. The research question was formulated as follows:

*How has the COVID pandemic affected e-commerce behavior in the Czech Republic?*

This research question is assessed from two points of view. The first one is represented by consumer activities, namely, the numbers of posts, likes, and shares. The second one analyzes the trends and differences in corporate activities by means of the numbers of posts.

**Figure 1:**
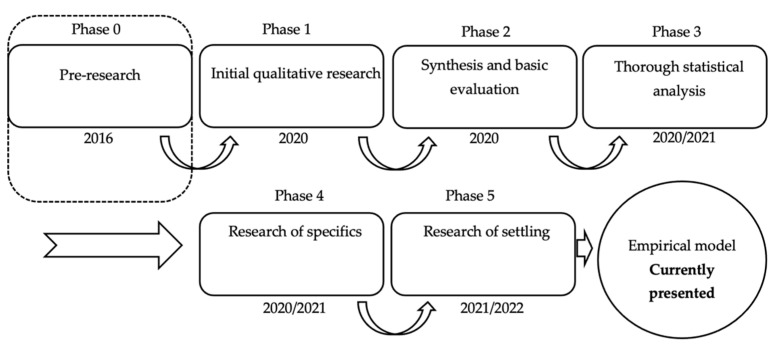
Research phases. Source: authors’ own processing based on [47].

From the point of view of the genesis of the research (presented in the Figure 1), this study is the output of Phase 5 of the research.

The empirical material necessary to create the model was collected through three measurements carried out in the following periods:

(a) First observation: from 12 March 2020 to 17 May 2020,
(b) Second observation: from 27 November 2020 to 14 January 2020,
(c) Third observation: from 12 March 2021 to 17 May 2021.

The research was conducted by means of monitoring Facebook activity on the official profiles/fan pages of the 5 largest Czech e-shops, which were ranked based on the order published by the online journal Ecommerce Bridge [48]—specifically, the profiles of Alza, Mall, CZC, Aukro, and Lidl Czech Republic. The research sample thus consisted of more than 1.5 million users with the following structure (see Table 1).

Data were collected daily for thirteen months by the interested researchers, who recorded the number, type, and nature of user interactions, as well as the number and nature of contributions/posts/publications on the profiles of the selected e-commerce entities.

The interactions of customer groups (for the purposes of the analysis) represented the reactions of individuals to e-marketing communications/posts that the subject/profile manager of a e-commerce entity published during the monitored period on the official profile of the given company.

The reactions—in our case, referred to as user interactions—had the forms of comments, likes, and sharing. The analysis was processed in several stages, or parts, using the following mathematical–statistical methods, including:

The Shapiro–Wilk test for normal distribution testing:

(1) $$SW=\frac{{\left(\sum u_{i}x_{i}\right)}^{2}}{\sum u_{i}^{2}\sum {\left(x_{i}-\bar{x}\right)}^{2}}$$

where: *u_i_*—constant;

*x_i_*—value of the i-th statistical unit;

$\bar{x}$—average value.

The Kruskal–Wallis test for testing the mean value differences between multiple samples:

(2) $$Q=\frac{12}{n\left(n-1\right)}\overset{I}{\underset{i=1}{\sum }}\frac{T_{i}^{2}}{n_{i}}-3\left(n+1\right)$$

where: *n*—number of observation periods;

*n_i_*—number of observation periods in the *i*-th group;

$T_{i}^{2}$—total sum of ranks in the *i*-th group.

The Mann–Whitney test for testing the mean value differences between two samples:

(3) $$U^{\prime }=n_{y}n_{x}\frac{n_{y}\left(n_{y}+1\right)}{2}-R_{y};U=n_{y}n_{x}-U^{\prime }$$

where: *n_x_*—number of observation periods, or the extent of the *x*-th sample;

*n_y_*—number of observation periods, or the extent of the *x*-th sample;

*R_y_*—sum of the order of the *y*-th file;

*U*, *U*′—test statistics.

The Levene test for homoskedasticity testing:

(4) $$LE=\frac{\left(N-k\right)}{\left(k-1\right)}\frac{\sum_{i=1}^{k}N_{i}{\left(Z_{i}-Z_{..}\right)}^{2}}{\sum_{i=1}^{k}\sum_{j=1}^{N_{i}}{\left(Z_{ij}-Z_{i.}\right)}^{2}}$$

where: *k*—number of values in the monitored variable category;

*N*—total number of observation periods;

*N_i_*—number of observation periods in the *i*-th group;

*Y_i_*_j_—measured value of the *j*-th unit of the *i*-th group;

$\bar{Y_{i}}$—average value of the *i*-th group;

${\tilde{Y}}_{i}$—median of the *i*-th group;

*Z*_.._—average of the groups *Z_ij_*;

*Z_i_*_._—average of *Z_ij_* for the *i*-th group.

The Kolmogorov–Smirnov test for testing the compliance of distribution functions:

(5) $$\begin{array}{cc} D_{n_{1},n_{2}}= & sup\left|F_{1,n_{1}}\left(x\right)-F_{2,n_{2}}\left(x\right)\right|\\ & -\infty <x<\infty \end{array}$$

where: $F_{1,n_{1}}\left(x\right)$—empirical distribution function of the first sample;

$F_{2,n_{2}}\left(x\right)$—empirical distribution function of the second sample.

A simple regression analysis using the Ordinary Least Squares (OLS) method was used to compare developments over time. It was verified by the coefficient of determination:

(6) $$R^{2}=\frac{\sum_{i=1}^{n}{\left(y_{i}-{\hat{y}}_{i}\right)}^{2}}{\sum_{i=1}^{n}{\left(y_{i}-{\bar{y}}_{i}\right)}^{2}}$$

where: $y_{i}$—measured value of the dependent variable;

${\hat{y}}_{i}$—estimated value of the dependent variable;

${\bar{y}}_{i}$—average value of the dependent variable.

The above mathematical–statistical methods were used for the verification of the partial results of the research. The main output of the research was a complex parameter that was identified as an engagement ratio; the parameter was calculated as follows:

(7) $$ER=\frac{{\tilde{L}}_{norm}+{\tilde{C}}_{norm}+{\tilde{S}}_{norm}}{{\tilde{P}}_{norm}}$$

where: ${\tilde{L}}_{norm}$—standardized mean value (median) of the number of likes within one observation;

${\tilde{C}}_{norm}$—standardized mean value (median) of the number of comments within one observation;

${\tilde{S}}_{norm}$—standardized mean value (median) of the number of shares within one observation;

${\tilde{P}}_{norm}$—standardized mean value (median) of the number of posts within one observation.

Due to the different ranges of the variables, outliers, or the absence of a normal distribution, margin normalization was used for the engagement ratio (*ER*):

(8) $$y_{ij}=\frac{x_{ij}-\min\left(x_{j}\right)}{\max\left(x_{j}\right)-\min\left(x_{j}\right)}$$

The analysis and statistical evaluation that were implemented were processed in MS Excel, Statistica 13.4, and Statgraphics XVIII.

## 3. Results and Discussion

The processed analysis was performed at the level of individual e-commerce parameters. The processing consisted of (a) a comparison using selected moment characteristics and the related tests and (b) a trend comparison using the OLS regression model. Both analyses were performed separately for weekends and working days. The purpose of this double processing was to point out the absolute differences in the levels of e-commerce parameters between the observation periods and, consequently, their development over time.

### 3.1. Evaluation of Trends and Changes in the Number of Posts

**Figure 2:**
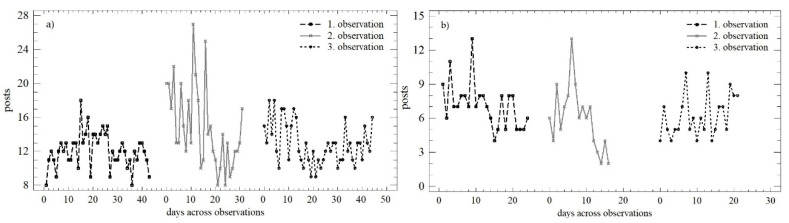
Scatterplot of the numbers of posts across observation periods: (**a**) working days; (**b**) weekend.

The development of the number of posts over time across the three observation periods is shown in Figure 2. Based on this graphical comparison, it is possible to consider the trend during the working days in the first and third observation as similar or identical. Higher variability is reported in the second observation, but this is attributed to the Christmas holidays. The trend during the weekend is continual, with a slight decrease in the number of posts in absolute terms.

The above trends were quantified by the OLS regression model, as described in Table 2. In the case of the number of posts, very similar trends could be observed over the working days and weekends. Between the first and second observations, there was a decrease in the daily increment, while the predictive value of each model increased.

**Figure 3:**
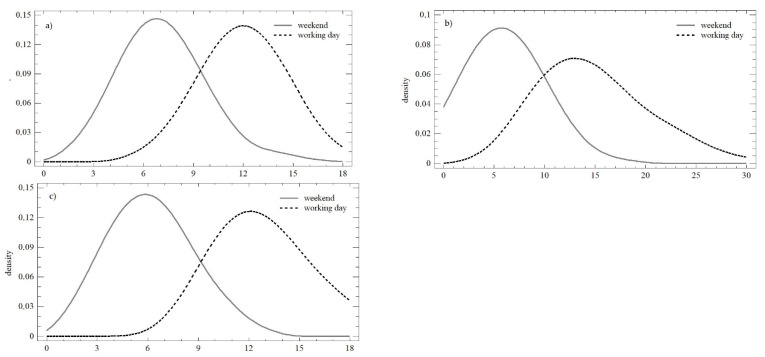
Density plot of the number of posts over the three monitored observation periods: (**a**) observation no. 1; (**b**) observation no. 2; (**c**) observation no. 3.

The differences in the two monitored parameters were confirmed as statistically significant for the number of posts during the working days (*LE* = 16.405; *p* < 0.01; *Q* = 6.064; *p* < 0.05); within the three observation periods, there was a change in the mean value, as well as the variance. The opposite trend was observed on weekends, which was in line with both the Levene test (*LE* = 1.167; *p* = 0.318) and the Kruskal–Wallis test (*Q* = 3.739; *p* = 0.154). Figure 3 shows the significant differences in the distribution functions during the individual observation periods. Differences were confirmed in all cases when comparing the mean values (W_obs1_ = 979; *p* < 0.01; W_obs2_ = 523.5; *p* < 0.01; W_obs3_ = 980; *p* < 0.01;), as well as the distribution functions themselves (K-S_obs1_ = 0.875; *p* < 0.01; K-S_obs2_ = 0.878; *p* < 0.01; K-S_obs3_ = 0.955; *p* < 0.01).

### 3.2. Evaluation of Trends and Changes in the Numbers of Comments

**Figure 4:**
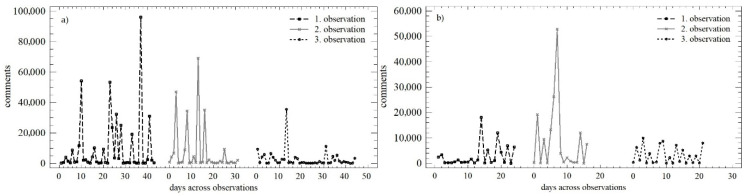
Scatterplot of the numbers of comments across the observation periods: (**a**) working days; (**b**) weekend.

The development of the number of comments over time within the three observation periods is shown in Figure 4. During the working days, the trend could be described as declining, with smaller absolute differences in the individually monitored days. On weekends, the homogeneity across the individual observation periods was higher, which negated the Christmas period recorded within the second observation. The differences in the numbers of comments during the individual days persisted, but were smaller in absolute terms.

The above trends could be quantified using the OLS regression model, which is described in Table 3. The number of comments showed almost the same development during the working days and weekends. There was a decrease in the daily increment, while the predictive value of individual models increased at the same time.

**Figure 5:**
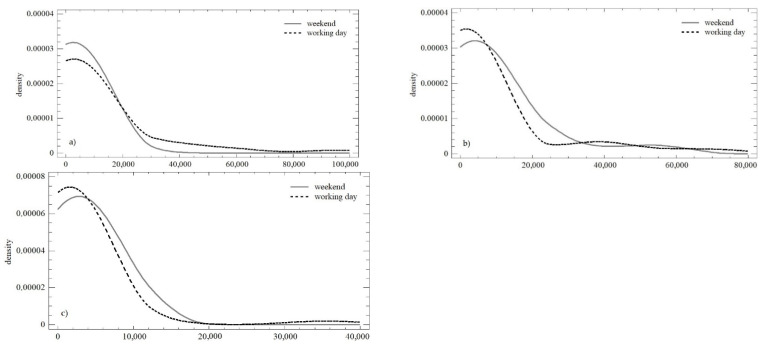
Density plot of number of comments over the three monitored observation periods: (**a**) observation no. 1; (**b**) observation no. 2; (**c**) observation no. 3.

During the working days, statistically significant differences in the mean value (median) were not confirmed (*Q* = 1.331; *p* = 0.513). However, differences in variance were confirmed (*LE* = 8.664; *p* < 0.01), which reflected the above-mentioned reduction in differences in daily values over time. Similar conclusions could be made for weekends (*LE* = 8.147; *p* < 0.01; *Q* = 2.532; *p* = 0.281). A comparison of the weekend and the working days within all three observation periods is presented in Figure 5. The distribution functions shown in each of the observation periods could be described as identical (K-S_obs1_ = 0.768; *p* = 0.596; K-S_obs2_ = 0.759; *p* = 0.611; K-S_obs3_ = 0.726; *p* = 0.667). The conformity could also be determined using the Mann–Whitney test and a comparison of the mean value.

### 3.3. Evaluation of Trends and Changes in the Numbers of Likes

**Figure 6:**
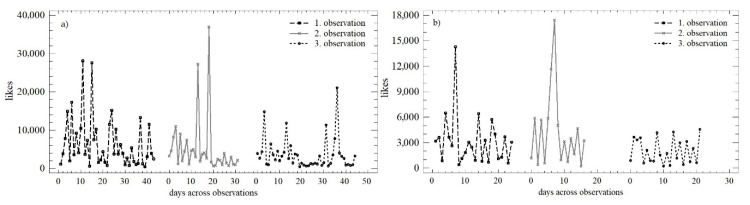
Scatterplot of the numbers of likes in the observation periods: (**a**) working days; (**b**) weekend.

Figure 6 shows the development of the number of likes over time within all three observation periods. During the working days, the trends were similar, with absolute differences in the individual monitored days that diminished over time. On weekends, the homogeneity in the first and the second observation periods was higher, which negate the Easter and Christmas periods recorded in these observation periods. The differences in the numbers of likes during the individual days persisted, but were smaller in absolute terms (especially in the third observation).

For the quantification the above-illustrated trends, the OLS regression model was used, which is presented in Table 4. In the case of the number of likes, we can see a very similar development during the working days and weekends. There was a continuous decrease in the daily increment, while the predictive value of the individual models increased (especially between the first and the second observations).

**Figure 7:**
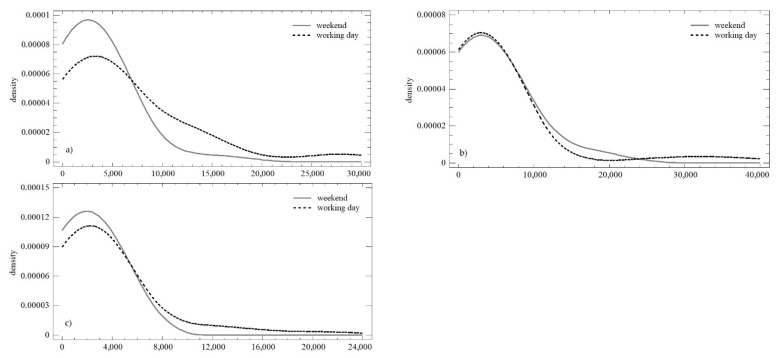
Density plot of the numbers of likes over the three monitored observation periods: (**a**) observation no. 1; (**b**) observation no. 2; (**c**) observation no. 3.

During the working days, statistically significant differences in the mean value (median; *Q* = 6.105; *p* < 0.05) and in the variance were confirmed (*LE* = 3.226; *p* < 0.05), which were affected by the above-mentioned holidays. During weekends, homoscedasticity was observed (*LE* = 3.585; *p* < 0.05), but the difference at the level of the mean value was confirmed again. (*Q* = 3.213; *p* = 0.200). A comparison of weekends and working days in all three observation periods is presented in Figure 7. Except for the first observation, the distribution functions could be described as identical (K-S_obs1_ = 0.349; *p* < 0.05; K-S_obs2_ = 0.176; *p* = 0.879; K-S_obs3_ = 0.231; *p* = 0.413). The conformity could not be confirmed using the Mann–Whitney test, i.e., there were significant differences between the first and second observation periods (W_obs1_ = 684; *p* < 0.05; W_obs2_ = 277; *p* < 0.05; W_obs3_ = 610; *p* = 0.126).

### 3.4. Evaluation of Trends and Changes in the Numbers of Shares

**Figure 8:**
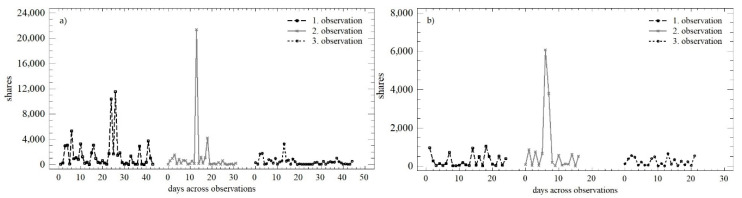
Scatterplot of the numbers of shares in the observation periods: (**a**) working days; (**b**) weekend.

The development of the number of shares over time within the three observation periods is shown in Figure 8. Based on this graphical comparison, it is possible to describe the trend during working days in the first and third observations as similar or identical. Higher variability was reported in the second observation, but this was attributed to the Christmas holidays. The trend during the weekend was continual, with a slight decrease in the number of shares in absolute terms.

The OLS regression model was used to quantify the above trends (see Table 5). In the case of the number of shares, a very similar development was seen during working days and weekends. There was a decrease in the daily increment, especially between the first and the second observations, while the predictive value of individual models slightly increased.

**Figure 9:**
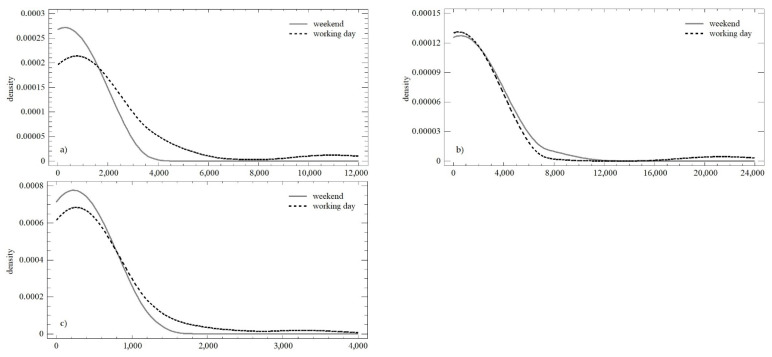
Density plot of the numbers of shares in three observation periods: (**a**) observation no. 1; (**b**) observation no. 2; (**c**) observation no. 3.

The differences in the two monitored parameters were confirmed as statistically significant for the number of shares during working days (*LE* = 4.097; *p* < 0.05; *Q* = 7.848; *p* < 0.05), i.e., in the three observation periods, there was a change in the mean value, as well as the variance. A partially opposite situation was recorded on weekends, where we found conformity at the level of the mean value (*Q* = 1.302; *p* = 0.521). The difference in the variance was confirmed (*LE* = 7.551; *p* < 0.01). Figure 9 shows the similarities in distribution functions during the individual observation periods (K-S_obs1_ = 0.395; *p* < 0.05; K-S_obs2_ = 0.141; *p* = 0.979; K-S_obs3_ = 0.177; *p* = 0.738). Differences at the level of the mean value were confirmed only in the first observation in all cases when comparing the mean values (W_obs1_ = 740.5; *p* < 0.01; W_obs2_ = 279; *p* = 0.891; W_obs3_ = 556; *p* = 0.419).

### 3.5. Delta of Changes—Engagement Ratio over the Observation Periods

The results of the above analysis performed at the level of individual e-commerce parameters are highly heterogeneous. The only phenomenon that occurred when monitoring the numbers of posts, comments, likes, and shares was a significant decrease in the trend level (regressor) of the regression model; see Table 6:

From the point of view of the absolute change in the mean value (Table 7), the monitored e-commerce parameters can be divided into two groups. The first group includes comments and shares whose trends during weekends and working days are the same (the decrease during working days is accompanied by a decrease during weekends). The second group consists of posts and likes in which a decline or stagnation is noticed regardless of the above division.

**Figure 10:**
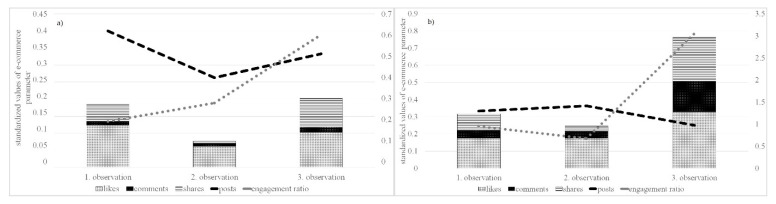
Engagement ratio and its components over individual observation periods: (**a**) working days; (**b**) weekends.

The effectiveness of the activities (posts) of the monitored institutions, considering the reactions in the forms of likes, comments, and shares, is shown using the engagement ratio in Figure 10. The trends monitored within the three observation periods were significantly different for weekends and working days. During the working days, after a significant decline in the second observation, a recovery in the third observation with greater customer activity in the form of shares was noticed. In the context of the change in the number of posts, the engagement ratio also doubled. During the weekends in the third observation, the interest of customers grew significantly, which was reflected in the significant growth of the engagement ratio.

**Figure 11:**
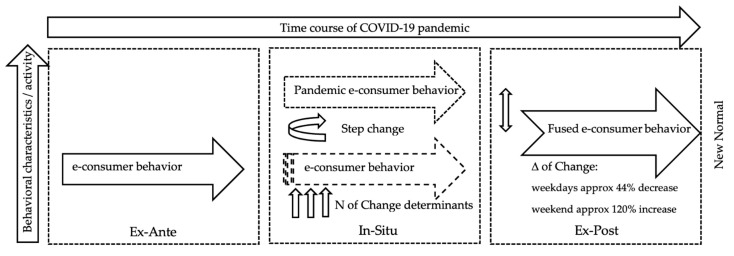
Empirical model of the impact of the COVID-19 pandemic on e-consumer behavior. Source: authors’ own processing based on [47].

Based on the presented findings, the research task can be considered to be solved. The individual partial analyses described in Section 4.1, Section 4.2, Section 4.3 and Section 4.4 provided the answer to the research question. Considering the comparison of the trend levels, significant changes were noticed in e-consumer behavior in all three observation periods—specifically, the decline in panic reactions recorded in the first observation and the subsequent stabilization of parameters in the form of a new normal. From the point of view of the effectiveness and management of the e-marketing communication of e-commerce entities, there was an increase in efficiency represented by the engagement ratio parameter, where the response of the target markets per unit of activity (post) slightly increased during working days. The return to the pre-crisis normal can especially be observed during weekends, where the values of user activity increased significantly throughout the observation periods. Users were thus partially returning to their pre-crisis weekend patterns of e-consumer behavior. A new normal was created by the synthesis of old and new patterns, as seen in Figure 11.

By answering the research question, it is possible to move smoothly on to the conclusion of the knowledge obtained based on the research.

## 4. Conclusions

This study synthesizes the results of knowledge in the field based on year-to-year comprehensive research, so we consider it important to summarize the results of both the final phase of research and the results of partial analyses on which the topic could be developed into its current and final form.

The first part of the conclusion summarizes the partial results of analyses published in a total of nine sub-studies in 2016, 2020, and 2021.

This part will be followed by the general conclusion, which concludes the research topic.

### 4.1. Evaluation of Partial Results

The starting point for the study of the issue was an empirical analysis [15] that examined the possibilities of effective use of the social network Facebook for the purposes of marketing communication by small and medium-sized enterprises. The study examined the interactions of companies and their customers during a model day and model week. The results provided relatively interesting information on the structuring of e-consumer behavior. Interactions of users (customers) with the marketing content of providers (companies) showed that, as a medium, the internet was already relatively well established in the pre-crisis period. In terms of specific findings, we confirmed that posts added over the weekend resulted in approximately 20% more interactions than posts added during the work week. Sharing interactions were at their highest at the beginning and end of the week. When it came to feedback in the form of comments, users maximized this interaction at the end of the work week. The working week as such was characterized by relatively average values of interactions. As for the model day, the interactions were at their highest in the early evening. As for the morning hours, we recorded more complex interactions only with average or below-average values. With these findings, we created a fundamental pattern of consumer behavior. In general, it can be stated that the domain of e-consumer activities in the pre-crisis period was the evening and weekends. This model corresponded to the generally accepted notion of online customer activity. In view of this finding, we decided not to develop the topic further.

With the onset of the global pandemic, e-consumer behavior has taken on a new dimension. The complexity of the topic was extensive, and the level of current knowledge was questionable. The market as such was on the verge of transformation. At this point, we decided to build on previous research and implement a series of empirical inquiries that were published as three conference papers that were, in principle, autonomous, and they were synthesized in a study [33] that provided the first more sophisticated insights into the topic being analyzed. For the purposes of the analysis, we examined user interactions in two similarly affected markets in Central Europe—the Slovak market and the market of the Czech Republic—during the first state of emergency caused by the COVID-19 pandemic. The market conditions were unprecedented, as the closure of the economy in both countries took an almost complete form that eliminated physical interactions. Using the same methodology, in the changed conditions of the first half of 2020, we came to relatively crucial findings regarding behavioral changes, reflecting the panic reaction of the market to the new, highly non-standard situation. Regarding user interactions for the marketing communications of the providers, we observed a fundamental regrouping of maximum interactions from both the perspective of the model week and the perspective of the model day. As for the model week, the maximums moved from the weekend to the middle of the working week on the Czech market, and at the beginning of the working week on the Slovak market. In both cases, the weekend became a dominant offline period. The maxima of interactions recorded during the working week showed signs of panic behavior. Selected interactions reached values of 200 to 450% above the weekly averages. As for the model day, the interactions were, again, fundamentally rearranged, reaching their maximum before noon. Thus, users moved their interactions to working time. This condition can be referred to as a pandemic condition. The final stage of the research of this phase was a study [32] in which we confirmed statistically significant changes in interactions during the working day and over the weekend. The work week, especially during the morning hours, literally exhausted the capacity of user interactions; on the other hand, weekends became domains for offline activities. This trend was strongly different from the pre-crisis period. From the point of view of the level of interactions and their maximums, we recorded this state as a state of e-consumer panic behavior.

In this phase of the research, we also tried to make the most of the relatively extensive feedback from the academic community, which was the direct personal feedback of the audience from scientific conferences or the well-meaning advice of reviewers of studies published in books and journals. The issue of seasonality and geographical specificity has emerged as crucial in terms of possible limitations. It is at this point that we have made every effort to minimize these limitations. The efforts were reflected in a study [47] in which we examined the seasonality aspect during the Christmas period of 2020/2021, as well as the geographical specificity of Central Europe (which experienced a relatively long period of major economic downturns caused by COVID-19) and the Baltic market, which, from the point of view of e-commerce players, is dominated by the multinational giants Amazon and eBay. The research confirmed the assumption of considerable regional specificity. The research also pointed to the factor of market development, as well as to the level of economic closure, which has proven to be a possible co-determinant of a shift in trends.

In any case, this stage provided us with starting points for further research. In the mentioned study, we presented a working version of the model and identified the key areas necessary for the analysis in order to finalize it. The working version of the model pointed to facts indicating a partial stabilization of trends in changes in e-consumer behavior, while due to the level and scope of processing of empirical material, it was not yet possible to proceed with the analyses necessary to verify the assumptions.

In any case, the accumulated knowledge base was sufficient for us to be able to formulate the assumption of the existence of a state that was marked as a new normal. We assumed that this was a condition that arose from a synthesis of the original pattern of behavior and the panic pattern of behavior. The present study was, therefore, devoted to quantifying the level of change, confirming the assumptions, and finalizing the research.

### 4.2. General Evaluation of Findings

E-commerce underwent a significant stress test at the time of the onset of the global COVID-19 pandemic. Panic buying, partial closure of brick-and-mortar shops, and the overall market uncertainty created unprecedented conditions for all market players. The dominant players on the supply side of the market were able to adapt in a relatively short time, as their digital infrastructure benefited from previous thorough optimization. Market players that were long hesitant to decide on whether and when to start with the digital transformation were forced to improvise under the pressure of market survival. The demand side of the market also faced its challenges, in particular, the management of its own time, which represented a largely complex element with the increasing interest in working from home. Early analyses [32,33] pointed to the paradox of maximizing e-commerce benefits for customers, who shifted the dominant part of their online activities to working days in order to make use of additional benefits. As the pandemic progressed, the panic of online customers stabilized, and the accumulation of online activities within the working morning spread smoothly throughout the working day. Weekends, on the other hand, became a time of digital relaxation. The massive drop in online interactions recorded during weekends compared to the pre-pandemic standard—captured in the reference research [15]—was largely taken by surprise by the operators of the supply side of the electronic market. They responded to this with a year-on-year decrease in online activity during weekends, while their online activity remained unchanged during working days. In terms of regional specificity, the findings pointed to the fact that the similarity of government restrictive measures combined with the level of development of the online market generated relatively equal market responses to the evolution of e-consumer behavior patterns [47]. They also pointed to the fact that, in terms of the e-market development parameter, the digital market in the Czech Republic showed significant signs of above-average development. As for the trends in the development of e-consumer behavior itself, as the pandemic progressed, the steepness of the trends began to decrease. Based on the deepening of knowledge, as well as on the preliminary processing of the data from the second observation period, it could be assumed that a unique trend of behavior would grow, which would be a synthesis of the pre-pandemic and panic reactions from the times of the first wave of the pandemic. This assumption was confirmed by the data from the third observation period, based on which the delta of change necessary to complete the empirical model was identified.

As we expected, the new normal took the form of a fusion of a pre-pandemic and a pandemic panic pattern. In this model market, this synthesis was expressed by the Δ of change. Specifically, there was a decrease in e-consumer interactions during the working week of about 44% and an increase in interactions over the weekend by about 120% (compared to a pandemic panic behavioral pattern). The trend of moving the maximum of interactions from the weekend to the working week persisted; in any case, in terms of weekends, those in the second year of the pandemic no longer presented their dominant offline nature. From this point of view, it is possible to state that there has been a partial return to the pre-crisis normal. Consumers are gradually adapting to the new state. With the adaptation comes the decline of relatively visible panic behavior from the first half of 2020. Panic behavior can be viewed on several levels. The first level is the panic of lack, and the second is the panic of consciously maximizing the benefits of one’s time. This phenomenon is especially visible in the shift of interactions across the model day. While, in the first months of the pandemic, users suddenly saturated their digital needs in the morning hours of the working day, as the pandemic progressed, they spread these activities over almost the entire working time. Simply put, this condition can be described as an imaginary loss of shyness. We are thus looking at maximizing the benefits of working time, where users/consumers are likely to maximize the benefits of distance work in the form of seeking sufficient benefits from consumption. As we did not notice significant differences in labor productivity, we assume that the time devoted to e-commerce activities has been replaced by consumers of traditional non-productive activities/social contacts, which they engaged in as employees during pre-pandemic working hours. However, we do not have sufficient empirical material to confirm this assumption. At this point, it can be said that the global pandemic is an evolutionary rather than a revolutionary element of change. However, in this case, it is an evolutionary element that is significantly accelerating the process of the natural transformation of systems in the transition from offline to online.

### 4.3. Empirical Implications for Science and Practice

At this point, the implications for science and practice were formulated. From the point of view of the deepening of knowledge in the field of e-commerce, it can be stated that the digital ecosystem is relatively resilient. Both sides of the market adapted relatively quickly to highly non-standard conditions, while the process of adaptation did not show significant shortcomings. The radical shift of the market from an offline to an online environment provides space for science to generate new knowledge. In terms of practical implications, it should be noted that both the demand and supply sides of the market actively used the circumstances to maximize the benefits. The supply side benefited from active feedback and, based on online information, actively optimized the process towards efficiency. This can be seen in the year-on-year decline in online activities during weekends, compared to an almost 120% increase in efficiency in the number of interactions per post. The demand side benefited from shortening communication channels, where it actively sought additional benefits from consumption in the form of discounts or promotions. These were a kind of reward for participating in the e-marketing communications of e-shops. Benefit maximization also occurred secondarily, where customers found the time needed for such participation in the morning, during working days. However, this trend stabilized as the pandemic progressed, but certain elements of this trend will be reflected in the new norm that will occur in the post-pandemic period.

In general, it can be stated that a proactive approach to digitization on both the supply and demand side is a new norm. The issue of finding effective tools for optimizing e-business is therefore a promising area for further research in economics and management sciences.

### 4.4. Theoretical Contributions

Consumer behavior is a phenomenon that integrates the views and approaches of a broad portfolio of scientific disciplines. Since the days of production marketing, which, in principle, described the sales process on the basis of an elementary shortcoming, it has undergone significant development over the last century. In Henry Ford’s time, it was enough to deliver a product to the market, and a competitive advantage was achieved by increasing production capacity. This situation has remained on the market for a considerable time. Product marketing brought new practices, customers began to choose, and their views and preferences were increasingly taken into account by the market. The mass communication media—first radio, and later television—took care of the third evolutionary shift in the issue. The concept of sustainable marketing in combination with the internet, which, in the zero years of the twenty-first century, represented the gold standard of the issue, did not, of course, represent a final and unchanging state. Customers have embraced the benefits of digitization; in any case, the brick-and-mortar market was still the starting point for the majority. The academic community, as well as practitioners [49], have led controversies about the future of the market, the pitfalls and opportunities of digitization, and the digital profile of the model customer’s day. During this period, we conducted the first studies in order to contribute to the knowledge of the issue. We found that the media consumer profile of the model customer is largely replaced by the internet. Compared to traditional media, which allowed conscious disconnection from the source of information by turning off the receiver, the internet on users’ mobile phones allowed content producers a permanent producer–consumer connection. By analyzing the initial patterns of behavior [15], we identified the basic e-consumer habits, which, in the form of a model day and a model week, described how it is possible to effectively time marketing communications for maximum response. The contribution of the study was largely empirical; in any case, it was based on model consumers’ media exposure, and we saw the decline of traditional media, as well as the removal of so-called dark spots from the time when the market was dominated by television. We assumed that the state of accelerated digitization would continue. We subsequently returned to consumer behavior research with the advent of the global pandemic, with unprecedented market conditions literally creating model conditions for building on previous research. The basic precondition for the shift in knowledge has become the question of whether a pandemic will cause the effect of a black swan in the market, or whether its effects will have a revolutionary or evolutionary impact on consumer behavior as such. In the early stages of the research described in the study, we, as researchers, were more inclined to assume a revolutionary change factor. However, through a continuous analysis of the issue, we identified a state of gradual decline in terms of the development of the observed characteristics of consumer behavior. By confirming the partial return of selected characteristics towards pre-pandemic trends (albeit at a significantly reduced intensity), we came to the conclusion that the new trend of the pre-pandemic normal will take the form of a fusion of pre-pandemic and pandemic behavior patterns. We recorded and confirmed another evolutionary shift in the development of consumer behavior; this time, it is not a change in terms of the preferred medium, but on the contrary, as a medium at the culmination of the pandemic, the internet confirmed its role as mass media and the stability of digital infrastructure. The change in consumer behavior is visible in greater producer–consumer interactions, as well as in a higher acceptance of the digital normal, i.e., a higher acceptance of digital habits in the consumer’s life. Based on empirical knowledge, it is, therefore, possible to express the shift in knowledge of consumer behavior in three words, namely, the phrase “accelerated digital transformation” of an evolutionary nature. With this statement, we would also end the conclusion and proceed to describe the limitations of the study and the prospects for further research on the issue.

### 4.5. Research Limitations

Despite the considerable efforts of the authors, it can be stated that the submitted study still has some limitations, which result predominantly from the nature of the data. Despite the large datasets, the data are predominantly of a qualitative nature. Another limitation is a certain degree of geographical specificity of the analyzed market. However, given the relatively high level of market development, the same results can be expected in markets with moderately to more developed online infrastructures.

### 4.6. Future Direction of Research

The issue of the global pandemic and its effects has a broad research scope. In terms of research into behavioral change, the re-closure of countries and their traditional infrastructures, combined with the application of social distancing, has created conditions that are unparalleled in modern history. With the gradual end of the pandemic, it is possible to expect that the topic as such will become even more relevant. Although it is currently relatively difficult to predict the long-term effects of a pandemic, in any case, as researchers, we see room for continuous empirical analysis of the issue. Based on the findings that we recorded in the various stages of the presented research, we are of the opinion that trends and indications for possible directions within the research appear continuously. A systemic approach to the issue is proving to be one of the possible directions for examining this phenomenon of the third decade of the twenty-first century.

## Figures and Tables

**Table 1:** Structure of the research sample. Source: authors’ own processing based on [32].

Facebook Profile	Users/Fans
Alza.cz	264,865
Mall.cz	207,747
CZC.cz	232,694
Aukro.cz	110,201
Lidl Czech Republic	778,673
Total	1,594,180

**Table 2:** Linear regression model for posts during working days and weekends over each observation.

Regression Models	
working days	O1: posts = exp (0.802651 * ln(days)) (R^2^ = 91.58)
	O2: posts = exp (0.646466 * ln(days)) (R^2^ = 99.79)
	O3: posts = exp (0.55297 * ln(days)) (R^2^ = 99.27)
weekends	O1: posts = exp (0.732627 * ln(days)) (R^2^ = 83.24)
	O2: posts = exp (0.476767 * ln(days)) (R^2^ = 90.30)
	O3: posts = exp (0.450476 * ln(days)) (R^2^ = 97.73)

* Are character for multiplication.

**Table 3:** Linear regression model for comments during working days and weekends in each observation.

Regression Models	
working days	O1: comments = exp (2.44449 * ln(days)) (R^2^ = 87.23)
	O2: comments = exp (1.77242 * ln(days)) (R^2^ = 94.16)
	O3: comments = exp (1.48921 * ln(days)) (R^2^ = 95.62)
weekends	O1: comments = exp (2.70824 * ln(days)) (R^2^ = 85.79)
	O2: comments = exp (2.17092 * ln(days)) (R^2^ = 93.07)
	O3: comments = exp (1.76516 * ln(days)) (R^2^ = 93.56)

* Are character for multiplication.

**Table 4:** Linear regression model for likes during working days and weekends in each observation.

Regression Models	
working days	O1: likes = exp (2.64873 * ln(days)) (R^2^ = 88.57)
	O2: likes = exp (1.9471 * ln(days)) (R^2^ = 97.94)
	O3: likes = exp (1.68417 * ln(days)) (R^2^ = 98.37)
weekends	O1: likes = exp (2.96765 * ln(days)) (R^2^ = 86.33)
	O2: likes = exp (2.2266 * ln(days)) (R^2^ = 97.50)
	O3: likes = exp (1.183614 * ln(days)) (R^2^ = 98.37)

* Are character for multiplication.

**Table 5:** Linear regression model for shares during working days and weekends in each observation.

Regression Models	
working days	O1: shares = exp (1.97912 * ln(days)) (R^2^ = 83.26)
	O2: shares = exp (1.33026 * ln(days)) (R^2^ = 91.65)
	O3: shares = exp (1.13619 * ln(days)) (R^2^ = 93.11)
weekends	O1: shares = exp (1.88783 * ln(days)) (R^2^ = 81.68)
	O2: shares = exp (1.54268 * ln(days)) (R^2^ = 90.11)
	O3: shares = exp (1.21997 * ln(days)) (R^2^ = 92.49)

* Are character for multiplication.

**Table 6:** Comparison of the trend level (regressor) of the regression model.

	Working Days	Weekends
posts	↓30.11%	↓38.51%
comments	↓39.07%	↓34.82%
likes	↓36.41%	↓60.11%
shares	↓42.59%	↓35.37%

**Table 7:** Comparison of the absolute changes in the mean value (median).

	Working Days	Weekends
posts	0%	↓21.42%
comments	↓47.55%	↑113.99%
likes	↓32.48%	↓41,17%
shares	↓51.57%	↑45.18%

## Data Availability

Not applicable.
